# TRIB3 inhibition by palbociclib sensitizes prostate cancer to ferroptosis via downregulating SOX2/SLC7A11 expression

**DOI:** 10.1038/s41420-024-02152-7

**Published:** 2024-10-03

**Authors:** Yangyi Zhang, Chenyu Liu, Yalan Yang, He Ren, Tianyi Ren, Yinuo Huang, Shinan Zhang, Qiang Sun, Hongyan Huang

**Affiliations:** 1 Department of Oncology, Beijing Shijitan Hospital of Capital Medical University, 10 TIEYI Road, Beijing, 10038 China; 2 Laboratory of Advanced Biotechnology, Beijing Institute of Biotechnology; Research Unit of Cell Death Mechanism, 2021RU008, Chinese Academy of Medical Science, Beijing, 100071 China

**Keywords:** Prostate cancer, Autophagy

## Abstract

Palbociclib is a CDK4/6 inhibitor approved for the treatment of breast cancer by suppressing cell proliferation. However, monotherapy with palbociclib was discouraging in prostate cancer, calling for a mechanism-based effective therapy. In this study, we reported in prostate cancer that palbociclib is a potent sensitizer of ferroptosis, which is worked out by downregulating the expression of TRIB3, a gene highly expressed in prostate cancer. Specifically, TRIB3 knockdown augmented the response of prostate cancer cells to ferroptosis inducers, whereas, TRIB3 overexpression rescued prostate cancer cells from palbociclib-induced ferroptosis. Mechanistically, TRIB3 inhibition by palbociclib resulted in downregulation of SOX2, which subsequently led to compromised expression of SLC7A11, a cystine/glutamate antiporter that counteracts ferroptosis. Functionally, a combined treatment of palbociclib with ferroptosis inducer significantly suppressed prostate cancer growth in a xenograft tumor model. Together, these results uncover an essential role of TRIB3/SOX2/SLC7A11 axis in palbociclib-induced ferroptosis, suggesting palbociclib a promising targeted therapy in combine with ferroptosis induction for the treatment of prostate cancer.

## Introduction

Prostate cancer (PCa) is one of the most common cancers worldwide, accounting for a large proportion of all cancer-related death [1, 2]. The treatment of prostate cancer have been greatly improved in the last decades, including radical prostatectomy, radiotherapy, and androgen deprivation therapy. Unfortunately, most patients experience relapse after initial therapy. Palbociclib is an oral small-molecule drug that works primarily by inhibiting the activity of cyclin-dependent kinases 4 (CDK4) and 6 (CDK6). CDK4 and CDK6 are key proteins in cell cycle regulation. They bind to the cell cycle D-type cyclin (Cyclin D) to promote the transition from the G1 phase to the S phase, thereby promoting cell division and proliferation [3]. This mechanism of action makes palbociclib an effective targeted therapy, providing patients with more treatment options [4, 5]. Previous studies have shown that combination with palbociclib could enhance the treatment of prostate cancer [6–8], but the functions and complexities of palbociclib in modulating the susceptibility of ferroptosis in prostate cancer requires further exploration.

Ferroptosis is a form of regulated cell death characterized by disruption of the antioxidative balance within cells [9–11]. During ferroptosis, there is an accumulation of ferrous iron and a decline in glutathione, which is crucial for regulating cellular redox balance. Additionally, ferroptosis disrupts cellular energy metabolism, evident by increased mitochondrial size coupled with decreased density and cristae. Although prostate cancer shows a certain level of sensitivity to ferroptosis, particularly in RB1-deficient cases, resistance under various conditions has been observed, limiting the effectiveness of ferroptosis inducers as a monotherapy [12–14]. The development of drugs that can enhance ferroptosis sensitivity by ferroptosis inducers represents for a promising strategy for clinical treatment.

In the current study, we discovered that palbociclib promotes ferroptosis susceptibility of prostate cancer LNCaP and PC3 cells, and TRIB3 was significant inhibited upon palbociclib treatment. Moreover, TRIB3 inhibition not only promotes cell cycle arrest but also ferroptosis in prostate cancer cells, whereas, overexpression of TRIB3 counteracts the ferroptotic response to palbociclib. Mechanistically, we identified that SLC7A11 was positively regulated by TRIB3 at the transcriptional level through the transcription factor SOX2.

## Results

### Palbociclib potentiates ferroptosis in prostate cancer cells

**Fig. 1:**
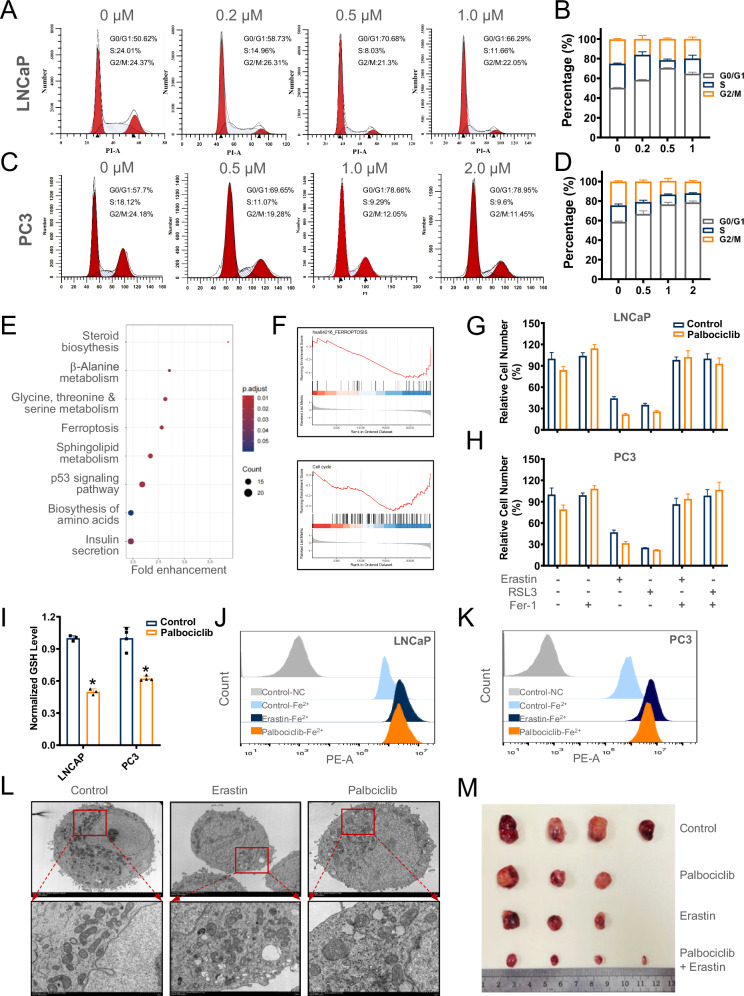
Palbociclib induces cell cycle arrest and promotes ferroptosis in prostate cancer cells. **A**–**D** The cell cycle of LNCaP and PC3 cells was detected after palbociclib treatment in different concentrations for one week. **E**, **F** The KEGG pathway and GSEA enrichment of RNA-Seq analysis in palbociclib-treated LNCaP cells. **G**, **H** The susceptibility of ferroptosis inducers was detected by CCK-8 assays in palbociclib-treated LNCaP and PC3 cells upon erastin, RSL3, and Fer-1. **I** The glutathione level, **J**, **K** ferrous iron levels were evaluated in LNCAP and PC3 cells with or without palbociclib treatment. **L** The morphological change analysis of mitochondria of LNCaP cells with either erastin or palbociclib treatment. **M** Representative images of the tumor size of cell line-derived xenografts (CDXs) of LNCaP cells. The nude mice were randomly divided into four groups including the palbociclib treatment group(70 mg/kg body weight), the erastin treatment group (20 mg/kg body weight), the combination treatment group, and the SD group for control. The above data are presented as the mean ± S.D. of at least three independent experiments. *P < 0.05, **P < 0.01.

To assess the impact of palbociclib on prostate cancer cells, cells were treated with palbociclib of different concentrations. As shown in Fig. 1A–D, 0.5 μM and 1 μM could effectively induce G1 phase arrest in LNCaP and PC3 cells, respectively, and therefore were selected for subsequent experiments. RNA-seq analyses revealed that ferroptosis was activated upon palbociclib treatment among the KEGG pathway (Fig. 1E). GSEA analysis indicated an enrichment of genes associated with cell cycle arrest and a reduction in the expression of genes that confer ferroptosis resistance in the palbociclib-treated cells (Fig. 1F).

We therefore examined ferroptosis in palbociclib-treated cell lines. As is shown in Fig. 1G, H, palbociclib could enhance ferroptosis inducers erastin and RSL3 cytotoxic effects in LNCaP and PC3 cells, besides, the ferroptosis inhibitor Ferrostatin-1(Fer-1) could reverse these effects. It is supposed that palbociclib may promote ferroptosis sensitivity in prostate cancer cells. To clarify whether palbociclib disrupts the antioxidative balance in prostate cancer cells, glutathione (GSH) levels were examined and a marked GSH decrease was detected upon palbociclib treatment (Fig. 1I). Meanwhile, FerrOrange staining assays demonstrated a dramatic increase of ferrous iron content in both LNCaP and PC3 cells treated with palbociclib (Fig. 1J, K). Consistently, palbociclib induced a morphological change in mitochondria as erastin did, including increased size, reduced membrane density, and thinning of cristae (Fig. 1L), which is in good agreement with ferroptosis characteristics. To further validate a role of palbociclib in promoting ferroptosis susceptibility in vivo, NCG mice bearing LNCaP cells were treated with palbociclib and erastin, alone or in combination. As shown in Fig. 1M, palbociclib treatment significantly potentiated the tumor-suppressive effect of erastin. Together, these results indicated that palbociclib could sensitize prostate cancer cells to ferroptosis both in vitro and in vivo.

### TRIB3 inhibition by palbociclib mediates cell cycle arrest in prostate cancer cells

**Fig. 2:**
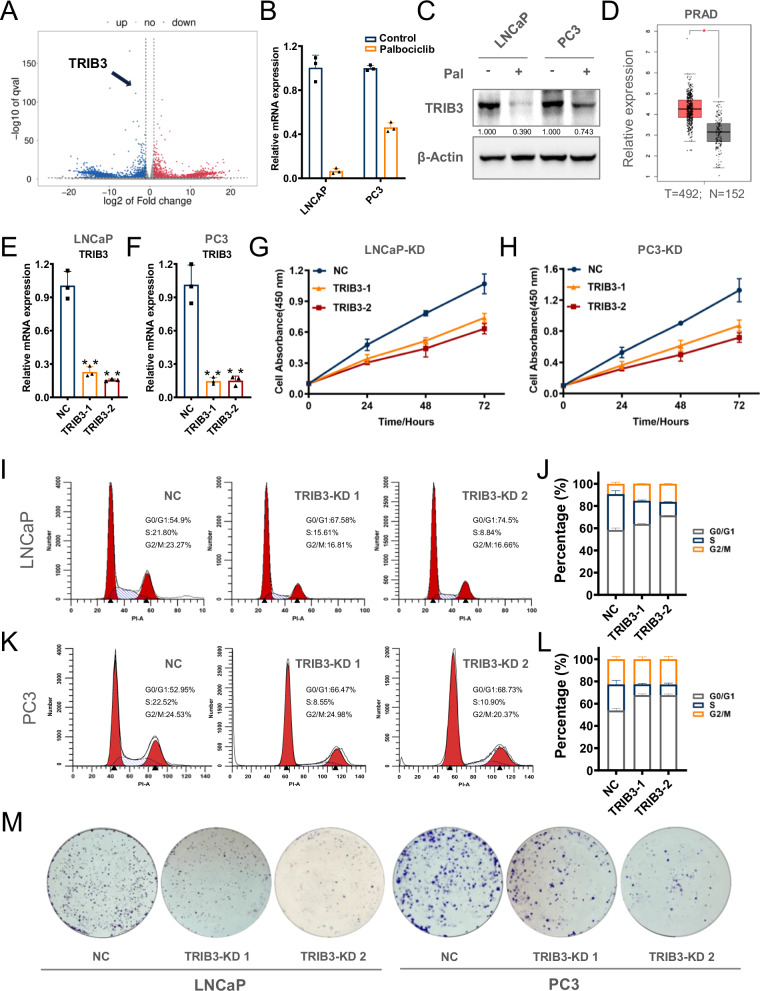
Palbociclib-induced TRIB3 inhibition is involved in the progression of cell cycle arrest. **A** The volcano plot elucidates the differential gene expression between palbociclib treatment and the counterpart of LNCaP cells, blue and red represent decreased and increased genes respectively. **B**, **C** qRT-PCR assays and western blotting assessed the mRNA and protein level of TRIB3 in LNCaP and PC3 cells with palbociclib treatment. **D** The expression of TRIB3 in prostate cancer patients from TCGA database (GEPIA, http://gepia.cancer-pku.cn/). **E**, **F** The efficiency of TRIB3 knockdown in LNCaP and PC3 cells was detected by qRT-PCR assays. **G**, **H** The Cell proliferation, **I**–**L** cell cycle, and **M** colony formation assays were analyzed both in LNCaP and PC3 TRIB3-KD cells.

To investigate the molecular mechanism underlying palbociclib-induced ferroptosis, gene expression profiling was performed on palbociclib-treated cells, which identified TRIB3 as one of the most downregulated genes in both LNCaP and PC3 cells (Fig. 2A–C). Notably, TRIB3 levels were found to be elevated in prostate cancer patients (Fig. 2D, *P* < 0.05), to explored the role of TRIB3 in cell cycle control of prostate cancer cells, two stable TRIB3 knockdown (KD) cell lines were generated for both LNCaP and PC3 cells using short hairpin RNA (shRNA) (Fig. 2E, F). As a result, the viability of KD-TRIB3 cell lines decreased by approximately 30% and 45% in LNCaP and PC3 cells, respectively, accompanied by a G1 phase arrest and a significant reduction in the S phase (Fig. 2I–L). Consistently, colony formation was significantly compromised upon TRIB3 knockdown in prostate cancer cells (Fig. 2M), suggesting an essential role of TRIB3 in prostate cancer.

**Fig. 3:**
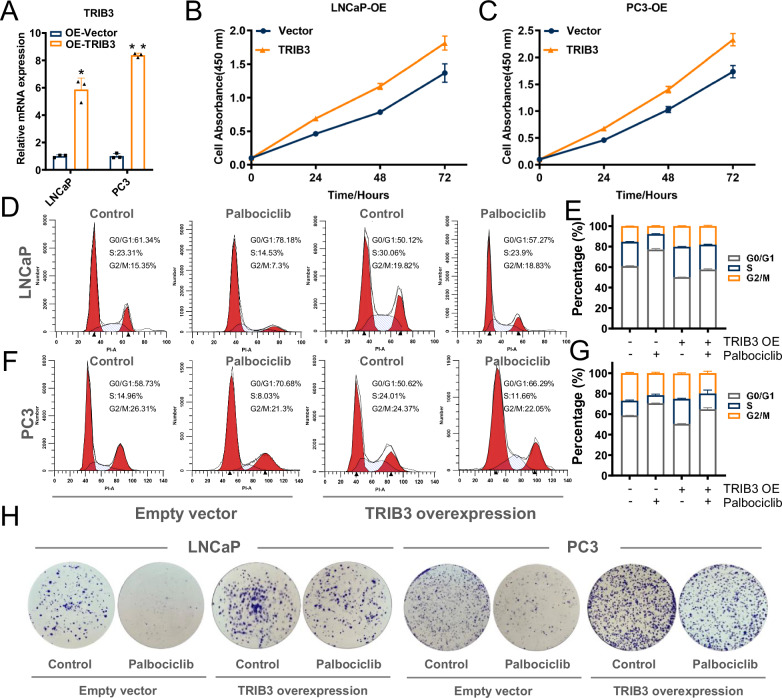
TRIB3 overexpression neutralizes the inhibition of cell growth by palbociclib in prostate cancer cells. **A** qRT-PCR assays detected the efficiency of TRIB3 overexpression in LNCaP and PC3 cells. **B**, **C** The cell proliferation in TRIB3 overexpression LNCaP and PC3 cells was evaluated by CCK-8 assays. **D**–**G** The cell cycle and **H** the colony formation assays were taken to detect the TRIB3 overexpression and Vector cells of LNCaP and PC3 cells with or without palbociclib treatment. The above data are presented as the mean ± S.D. of at least three independent experiments. *P < 0.05, **P < 0.01.

To determine whether TRIB3 mediates the inhibitory effects of palbociclib on the growth of prostate cancer cells, we ectopically expressed TRIB3 in LNCaP and PC3 cells (Fig. 3A). CCK-8 assays indicated that TRIB3 overexpression increased cell proliferation (Fig. 3B, C). Consistently, overexpressed TRIB3 (OE-TRIB3) increased the proportion of S phase, and was able to rescue cell cycle arrest induced by palbociclib in both lines of cells (Fig. 3D–G). In addition, colony formation increased upon TRIB3 overexpression, indicating a resistance to palbociclib-induced growth inhibition (Fig. 3H). Together, these results suggested that TRIB3 is a pivotal target of palbociclib, which was involved in cell cycle control and cell proliferation in prostate cancer cells.

### TRIB3 inhibition by palbociclib promotes ferroptosis in prostate cancer cells

**Fig. 4:**
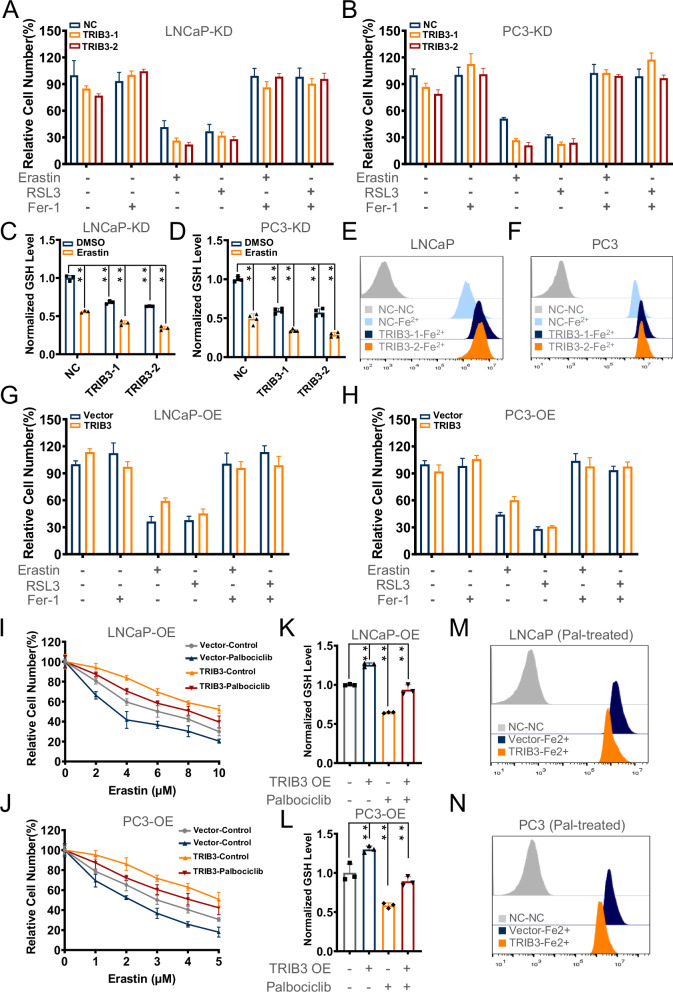
TRIB3 inhibition of palbociclib triggers ferroptosis in prostate cancer cells. **A**, **B**, **G**, **H** CCK-8 assays evaluated the susceptibility of erastin and RSL3 in LNCaP and PC3 TRIB3-KD and TRIB3-OE cells, Fer-1 as a specific ferroptosis inhibitor. **C**, **D** The GSH level and **E**, **F** the ferrous iron level were detected after erastin treatment for PC3 and LNCaP TRIB3-KD cells and control cells. **I**, **J** The sensitivity of erastin, **K**, **L** the GSH level, and **M**, **N** the ferrous iron level were analyzed in LNCaP and PC3 TRIB3-KD cells with palbociclib treatment. the above data are presented as the mean ± S.D. of at least three independent experiments. *P < 0.05, **P < 0.01. TRIB3-KD means knockdown TRIB3, TRIB3-OE means TRIB3 overexpression.

In order to explore whether TRIB3 is involved in ferroptosis sensitivity enhancement by palbociclib, we treated LNCaP and PC3 KD&OE-TRIB3 cells with ferroptosis inducers and inhibitor. As depicted in Fig. 4A, B, TRIB3 knockdown seems to enhance the cytotoxic effects of erastin, an effect that could be reversed by Fer-1. Concomitantly, TRIB3 knockdown cells exhibited a reduction in GSH levels, further exacerbated by erastin (Fig. 4C, D). FerrOrange staining assays showed a higher ferrous iron content in TRIB3 knockdown cells treated with erastin (Fig. 4E, F). Conversely, it is supposed that TRIB3 overexpression mitigated the toxicity of ferroptosis inducers on prostate cancer cells, especially reducing erastin’s cytotoxic effect (Fig. 4G, H). To further determine whether TRIB3 plays a role in palbociclib-induced susceptibility to ferroptosis, we assessed the sensitivity of TRIB3 overexpression cells to erastin, with or without prior palbociclib treatment. The results appeared that TRIB3 overexpression decreased sensitivity to erastin, which was prominently inhibited by a pre-palbociclib treatment (Fig. 4I, J). Moreover, TRIB3 overexpression cells showed higher GSH levels and enhanced antioxidative capacity, maintaining relatively stable GSH and ferrous iron levels upon palbociclib treatment (Fig. 4K–N). In conclusion, these findings suggested that TRIB3 inhibition by palbociclib could increase the susceptibility of prostate cancer cells to ferroptosis.

### TRIB3 increases SLC7A11 transcription via SOX2

**Fig. 5:**
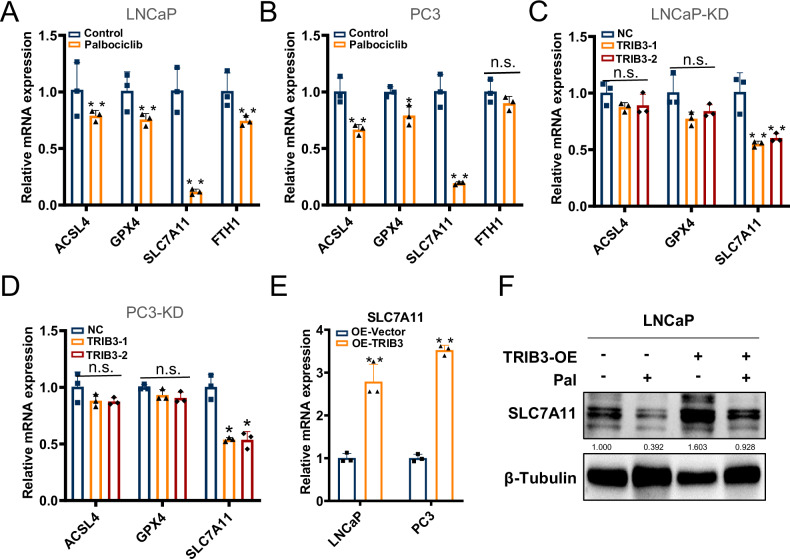
Palbociclib-treated TRIB3 inhibition decreased the expression of SLC7A11. Ferroptosis-related genes were detected in **A**, **B** palbociclib treatment, and **C**, **D** TRIB3-KD LNCaP and PC3 cells. **E** the expression of SLC7A11 was evaluated in LNCaP and PC3 TRIB3-OE cells by qRT-PCR assays. **F** The rescue assay for protein level alteration of SLC7A11 was evaluated in LNCaP TRIB3-OE cells and empty vector control cells with or without palbociclib treatment. The data are presented as the mean ± S.D. of at least three independent experiments. *P < 0.05, **P < 0.01.

To explore the molecular mechanisms downstream TRIB3 that mediates ferroptosis induced by palbociclib in prostate cancer cells, we examined the expression of ferroptosis-related genes upon palbociclib treatment. As depicted in Fig. 5A, B, palbociclib treatment led to the downregulation of several ferroptosis-related genes, including ACSL4, GPX4, and SLC7A11. Coincidently, it seems that TRIB3 knockdown (KD-TRIB3) caused reduction of SLC7A11, a membrane channel critical for ferroptosis inhibition (Fig. 5C, D), whereas overexpression of TRIB3 could increase the expression of SLC7A11 (Fig. 5E, F). These data indicated that TRIB3 positively regulated SLC7A11 expression in prostate cancer cells.

**Fig. 6:**
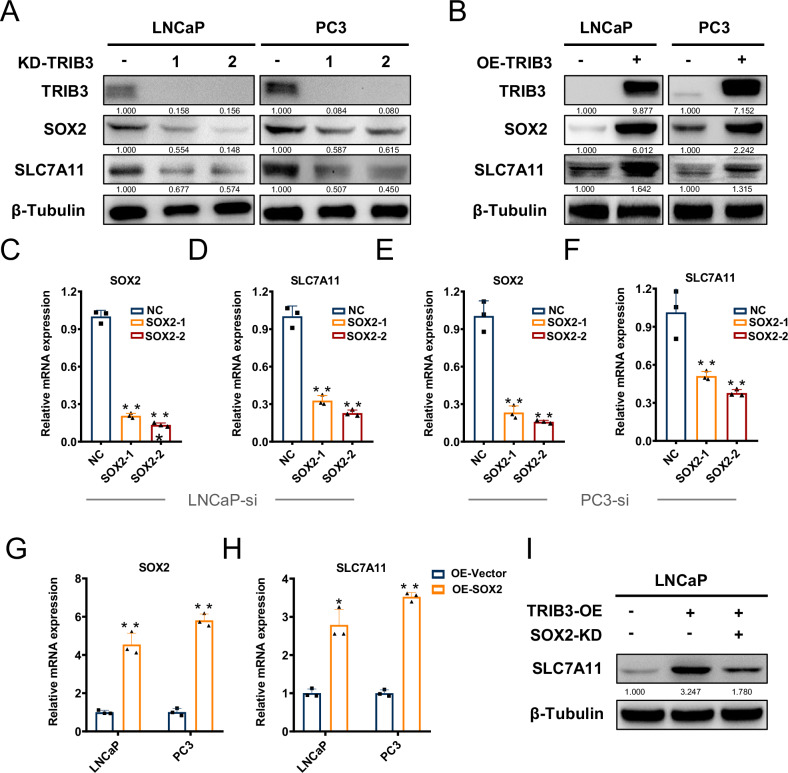
TRIB3 increases SLC7A11 transcription via SOX2 expression enhancement. **A**, **B** Western blotting assay detected the protein level of TRIB3, SOX2, and SLC7A11 in LNCaP and PC3 TRIB3-KD/TRIB3-OE cells. **C**–**H** The expression of SOX2 and SLC7A11 in si-SOX2 and oe-SOX2 cells of LNCaP and PC3 cells. **I** The rescue assay evaluated the protein level alteration after palbociclib treatment in LNCaP TRIB3-OE cells.

TRIB3 was reported to be able to regulate the expression of SOX2, a transcriptional factor potentially targeting SLC7A11 [15, 16]. We therefore explore a role of SOX2 in regulating SCL7A11 by TRIB3. Western blotting revealed that KD-TRIB3 led to a decrease in SOX2 and SLC7A11 expression, while overexpression of TRIB3 may increase their levels (Fig. 6A, B). Furthermore, the expression of SLC7A11 decreased upon SOX2 knockdown (Fig. 6C–F), whereas overexpression of SOX2 could upregulate SLC7A11 expression (Fig. 6G, H). Rescue experiments showed that TRIB3 could enhance the expression of SLC7A11, while this effect was reversed by SOX2 inhibition (Fig. 6I).

## Discussion

**Fig. 7:**
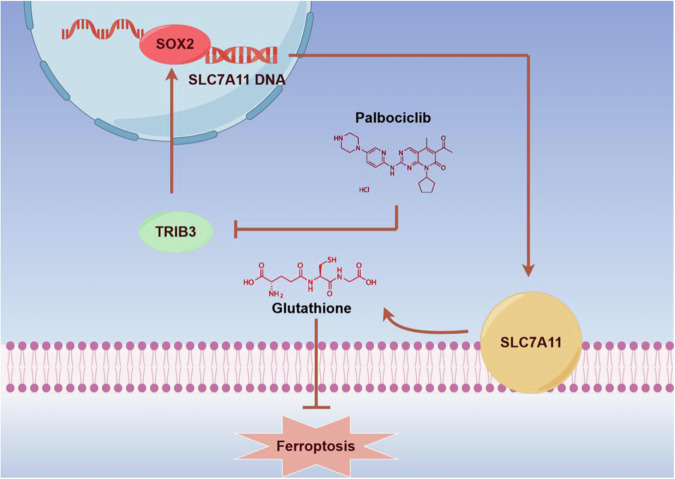
The schematic diagram showed that palbociclib induces ferroptosis in prostate cancer cells by inhibiting the expression of TRIB3, thereby reducing SLC7A11 expression via the TRIB3/SOX2/SLC7A11 pathway. Drawing by Figdraw.

Previous studies indicated that CDK4/6 inhibitors could induce cell cycle arrest and increase the ferroptosis sensitivity of cancer cells through lipid metabolic process disruption [17–19]. Our study found that palbociclib affects the cellular iron and GSH content through TRIB3/SOX2/SLC7A11 signaling axis, disrupting the cellular redox balance and promoting ferroptosis in prostate cancer cells (Fig. 7). We found that prostate cancer cells treated with palbociclib exhibited molecular alterations similar to those observed during ferroptosis, including reduced GSH level and increased ferrous iron content. The application of ferroptosis inducers, erastin, and RSL3, induced stronger ferroptosis in palbociclib-treated LNCaP and PC3 cells, indicating increased ferroptosis susceptibility by palbociclib. This susceptibility could be mitigated by the specific ferroptosis inhibitor Fer-1. Moreover, combining palbociclib with erastin significantly inhibited prostate cancer growth in vivo, providing a novel potential therapeutic approach for prostate cancer in clinics.

Previous studies have demonstrated that TRIB3 was oncogenic across various cancer types, characterized by stemness formation, the impediment of autophagic and proteasomal degradation processes, and cancer immune evasion [20–24]. In our research, we observed a pronounced overexpression of TRIB3 in prostate cancer tissues, which significantly promotes cell proliferation and colony formation. This effect is associated with increased S phase in LNCaP and PC3 cells. Additionally, our findings suggest a critical role of TRIB3 in the modulation of ferroptosis, where inhibiting TRIB3 activity potentiates the sensitivity of cancer cells to ferroptosis inducers, such as erastin, which is correlated with reduced ferrous iron and GSH levels. Conversely, TRIB3 overexpression conferred resistance to erastin in prostate cancer cells, highlighting a potential therapeutic target. Interestingly, the overexpression of TRIB3 appeared to counteract the palbociclib-induced G1 phase arrest and the associated decline in colony formation in prostate cancer cells. Cells overexpressing TRIB3 (OE-TRIB3 cells) exhibited enhanced resistance to ferroptosis induced by palbociclib, manifesting as higher levels of glutathione, less accumulation of ferrous iron, and diminished responsiveness to erastin upon palbociclib treatment. These results suggest that palbociclib may exert its anti-cancer effects, in part, through the suppression of TRIB3 expression, thereby inducing ferroptosis in prostate cancer cells.

The cystine transporter SLC7A11 plays a pivotal role in tumor growth promotion. Acting as an executor that uptakes cystine and excretes glutamate, disrupting SLC7A11 expression can lead to cellular ferroptosis [25–28]. In our study, we observed a suppression in the expression of several ferroptosis-related genes following treatment with palbociclib, particularly SLC7A11. Further investigation revealed that TRIB3 knockdown reduced SLC7A11 transcription levels, whereas its expression was augmented in TRIB3-overexpressing (OE-TRIB3) cells. Through rescue assays, we demonstrated that TRIB3 is implicated in the palbociclib-induced suppression of SLC7A11. Our results indicated that SLC7A11 levels decreased upon palbociclib treatment but remained elevated in OE-TRIB3 LNCaP cells irrespective of palbociclib treatment. Previous studies have identified the stem cell factor SOX2 as a transcription factor for SLC7A11 [16, 29–31], with further research suggesting that TRIB3 may regulate SOX2 expression [15, 32]. However, there is no evidence for the regulation relationship between TRIB3 and SOX2 in prostate cancer. We confirmed that TRIB3 overexpression increased SOX2 protein levels, whereas SOX2 expression was reduced in TRIB3 knockdown (KD-TRIB3) cells. Additionally, SLC7A11 expression decreased in SOX2 knockdown LNCaP and PC3 cells but was increased in SOX2 overexpression cells. Notably, knocking down SOX2 in OE-TRIB3 LNCaP cells reversed the TRIB3-induced upregulation of SLC7A11. Collectively, these findings suggest that palbociclib induces ferroptosis in prostate cancer cells by inhibiting the expression of TRIB3, thereby reducing SLC7A11 expression via SOX2. Our research underscores the potential of combining palbociclib with erastin as a therapeutic strategy to sensitize prostate cancer cells to ferroptosis, offering a novel option for the treatment of prostate cancer.

## Method

### Cell culture and treatment

The human prostate cancer cell lines PC3 cell line was obtained from the Cell Resource Center, Peking Union Medical College (PCRC) and the LNCaP cell lines were a gift from the Tianjin Institute of Urology. Cells were checked for mycoplasma using the GMyc-PCR Mycoplasma Test Kit (Yeasen Biotechnology). The prostate cancer cells were cultured in RPMI 1640 (MACGENE Technology Ltd., Beijing, China) medium, and human 293 T cell lines were cultured in DMEM with 10% FBS (ExCell Bio). All cells were cultured in a 1% penicillin-streptomycin with the humidified incubator of 5% CO2 at 37 °C. Palbociclib was purchased from Selleck (S1116), and dissolved in sterile deionized water, the concentration of the storage solution is 10 mM, aliquoted and stored in −80 ultra-low temperature refrigerator, and used within three months.

### Cell colony formation, cell proliferation, and cytotoxicity assays

To evaluate the efficacy of cell proliferation of OE-TRIB3 and KD-TRIB3 in LNCaP and PC3 cells,1 × 10³ cells were seeded in 6-weel plates. After two weeks cultivation and washed with PBS, samples Stained with 0.1% crystal violet (Solarbio, G1064) for colony formation detection. The CCK-8 assay in accordance with the manufacturer’s instructions (APExBIO) was taken to evaluate the cell viability. 5 × 10³ cells were seeded in 96-well plates and cell metabolic activity, indicative of cell viability, was then assessed in every period of twenty-four hours. Cell cytotoxicity assays were similar to cell growth detection, after seeding in 96-well plates, cell-cultured with a concentration gradient of drugs for 48 h. Particularly, to evaluate the palbociclib-induced ferroptosis effect, cells were exposed to palbociclib for a week before ferroptosis inducers and inhibitor treatment.

### Cell cycle

1 × 10^6^ cells were collected and Fixed in 75% alcohol at −20 °C overnight, after centrifugation and washed with PBS,cells were incubation with RNase and then stain with PI according to Cell Cycle Assay Kits (Solarbio,CA1510) manuscription. Data were analyzed by the software Flowjo and ModfitLD.

### Western blot

Cells were lysed by RIPA buffer (Boster) with protease inhibitor and Phosphoprotease inhibitor cocktail. After ultrasound with an ultrasonic crusher and centrifuge, the supernatant was extracted. Protein concentration was determined with a BCA protein assay kit (Thermo Fisher). 30 μg proteins were separated by 10% sodium dodecyl sulfate-polyacrylamide gel electrophoresis (SDS-PAGE) and then transferred to nitrocellulose membranes. Antibodies with working dilution, company source, and catalog number are listed below: anti-TRIB3 (1:2000 for WB, Nature biosciences, A53412), anti-SOX2 (1:1000 for WB, ProteinTech; #11064-1-AP), anti-SLC7A11 (1:2000 for WB, ProteinTech; #26864-1-AP), anti-β-Tubulin (1:10000 for WB, ProteinTech; #66240-1-Ig).

### RNA isolation and quantitative real-time PCR (RT-PCR)

Total RNAs from cells were isolated with Trizol reagent (Sigma–Aldrich, #T9424) and were reverse-transcribed with the HiScript IV RT SuperMix for qPCR (Vazyme, #R423-01) according to the manufacturer’s instructions. Quantitative RT-PCR was performed routinely using target primers for the amplification of the coding region of the target gene using SYBR Green Realtime PCR Master Mix (TOYOBO, #QPK-201). β-actin was used as an internal control. The primer sequences are listed in Supplementary Table S1.

### RNA sequencing and bioinformatics analysis

Total RNA was isolated from LNCaP and LNCaP palbociclib-treated cells. The RNA sequencing was performed with Illumina HiSeq 6000 as instructed by the manufacturer (lc-bio, Hangzhou). Fastp software (https://github.com/OpenGene/fastp) was used to remove the reads that contained adaptor contamination, low-quality bases, and undetermined bases with default parameter. Then sequence quality was also verified using fastp. We used HISAT2 (https://ccb.jhu.edu/software/hisat2) to map reads to the reference genome of Homo sapiens GRCh38. The mapped reads of each sample were assembled using StringTie (https://ccb.jhu.edu/software/stringtie) with default parameters. Then, all transcriptomes from all samples were merged to reconstruct a comprehensive transcriptome using gffcompare (https://github.com/gpertea/gffcompare/). After the final transcriptome was generated, StringTie and was used to estimate the expression levels of all transcripts. StringTie was used to perform expression level for mRNAs by calculating FPKM (FPKM = [total_exon_fragments/mapped_reads(millions) × exon_length(kB)]). The differentially expressed mRNAs were selected with fold change >2 or fold change <0.5 and with parametric *F*-test comparing nested linear models (*p* value < 0.05) by R package edgeR (https://bioconductor.org/packages/release/bioc/html/edgeR.html). The expression of TRIB3 in prostate cancer patients from the TCGA database was evaluated by GEPIA(http://gepia.cancer-pku.cn/).

### Plasmids, siRNA, and transfection

The PCDH-TRIB3-Puro-3×Flag plasmid and control vector were obtained from Youbio. Oligonucleotides encoding short hairpin RNAs (shRNAs) targeting TRIB3 were cloned into the pLKO.1-Puro lentiviral vector. The overexpression (OE) and knockdown (KD) plasmids, along with the pMD2.G and psPAX2 packaging vectors, were transfected into 293 T cells using jetPRIME transfection reagent (Polyplus-transfection) to produce lentiviruses. An empty vector (EV) was used as a negative control. Following 24 h of lentiviral infection and subsequent 24-h culture, positive LNCaP and PC3 cell clones were selected using puromycin (2 μg/mL) for 48 h with two rounds of selection. Additionally, the genes SOX2 were cloned into the pcDNA3.1-3xFlag-C vector and introduced into cells using jetPRIME transfection reagent. Transient transfection of siRNAs was performed with Lipofectamine RNAiMAX Reagent (Invitrogen, #13778-150). The sequences for all shRNAs and siRNAs used in this study are provided in Supplementary Table S2.

### Intracellular iron content and glutathione (GSH) level

To measure the alteration of Fe^2+^ content in PCa cells, we harvested cancer cells and stained them with ferrous iron probe of Iron Assay Kit (JODINJO, F374). According to the manuscription, after staining 37 °C for 30 min, cells washed three times with phosphate-buffered saline (PBS) and then analyzed them using a flow cytometer’s phycoerythrin (PE) channel. The GSH content was detected using the Reduced Glutathione Assay Kit according to the manufacturer’s protocol (Nanjing Jiancheng, China).

### Transmission electron microscope (TEM)

A total of 1 × 10^6^ Cells were collected and fixed with 2.5% glutaraldehyde in 0.1 M phosphate-buffered glutaraldehyde. Ultra-structural images were captured with the. TEM images were captured with transmission electron microscope (JEM-1011, Japan) which conducted by Hangzhou Yanqu Information Technology Co., Ltd.

### Tumor xenograft model

A total of 3 × 10^6^ LNCaP cells were subcutaneously injected into the right flanks of 4-week-old male NCG mice (Gempharmatech, Co). Tumor burdens were closely monitored by tumor volumes. The nude mice were randomly divided into four groups when the average tumor volume reached ≈0.1 cm^3^. When the largest tumors reached a size of 1.0 cm3, all mice were sacrificed due to ethical considerations. Moreover, the final tumor weight was also recorded. (a) SD: control, gavage/intraperitoneal injection for the same amount of normal saline/DMSO plus corn oil; (b) Palbociclib treatment group: palbociclib was dilution in water at 10 mg/mL, and gavage every two days for 7 times The volume of palbociclib for each nude mouse was 70 mg/kg. (c) Erastin treatment group: intraperitoneal injection for mice with 20 mg/kg body weight in 20 μl DMSO plus 130 μl corn oil erastin, every three days; (d) Group of palbociclib combined with sequential Erstin: Taken palbociclib gavage for 4 times in the first 8 days, and subsequent erastin intraperitoneal injection for 2 times, the volume of each treatment was same above. For 14 days of continuous treatment and observation, the mice were sacrificed by CO2 carbon dioxide asphyxiation and cervical dislocation, the subcutaneous tumor was quickly peeled off. After rinsing with physiological saline, half of the tissue was cut and placed in liquid nitrogen, and the rest of the tissue was fixed in 4% paraformaldehyde solution and later embedded in paraffin wax to form paraffin sections. All animal experiments were approved by the Animal Ethics Committee at the Shijitan Hospital of Capital Medical University and performed in accordance with the relevant guidelines and regulations.

### Statistical analysis

All statistical analyses were performed using GraphPad Prism 9.0 (GraphPad Software, USA). The data are presented as the mean ± standard deviation (SD) from three independent experiments. Statistical significance was analyzed using Student’s *t*-test or two-way ANOVA tests. All data were considered statistically significant at a *p* value < 0.05 *, *p* value < 0.01 **.

## Supplementary information

Supplementary Table S1

Supplementary Table S2

Original Data File

## Data Availability

All data generated during this study are included in this article.
