# Supplementary material for: TRIB3 inhibition by palbociclib sensitizes prostate cancer to ferroptosis via downregulating SOX2/SLC7A11 expression
Source: Cell Death Discov. 2024 Oct 3;10:425. doi: 10.1038/s41420-024-02152-7 (PMC11450094; doi:10.1038/s41420-024-02152-7)
Supplement: Supplementary file 1 — Supplementary Table S1 [file 41420_2024_2152_MOESM1_ESM.pdf]

Supplementary Table 1. Primers used for gene expression analysis.

Primer	Forward (5’-3’)
TRIB3	AAGCGGTTGGAGTTGGATGAC
ACSL4	CATCCCTGGAGCAGATACTCT
GPX4	GAGGCAAGACCGAAGTAAACTAC
SLC7A11	GGTCCATTACCAGCTTTTGTACG
FTH1	TCCTACGTTTACCTGTCCATGT
SOX2	GCCGAGTGGAAACTTTTGTCG

Reverse (5’-3’)
CACGATCTGGAGCAGTAGGTG
TCACTTAGGATTTCCCTGGTCC
CCGAACTGGTTACACGGGAA
AATGTAGCGTCCAAATGCCAG
GTTTGTGCAGTTCCAGTAGTGA
GGCAGCGTGTACTTATCCTTCT
