# Supplementary material for: TRIB3 inhibition by palbociclib sensitizes prostate cancer to ferroptosis via downregulating SOX2/SLC7A11 expression
Source: Cell Death Discov. 2024 Oct 3;10:425. doi: 10.1038/s41420-024-02152-7 (PMC11450094; doi:10.1038/s41420-024-02152-7)
Supplement: Supplementary file 2 — Supplementary Table S2 [file 41420_2024_2152_MOESM2_ESM.pdf]

Supplementary Table 2. The shRNA and siRNA sequences used for silencing TRIB3 and SOX2.

shRNA&siRNA	Forward (5'-3')	Reverse (5'-3')
sh-TRIB3-1	GATCTCAAGCTGTGTCGCTTT	AAAGCGACACAGCTTGAGATC
sh-TRIB3-2	GCCGTGCTCTTCCGCCAGATG	CATCTGGCGGAAGAGCACGGC
si-SOX2-1	CUCAUGAAGAAGGAUAAGUTT	ACUUAUCCUUCUUCAUGAGCG
si-SOX2-2	GACAGUUACGCGCACAUGATT	UCAUGUGCGCGUAACUGUCCA
