# Supplementary figures and images for: TRIB3 inhibition by palbociclib sensitizes prostate cancer to ferroptosis via downregulating SOX2/SLC7A11 expression
Source: Cell Death Discov. 2024 Oct 3;10:425. doi: 10.1038/s41420-024-02152-7 (PMC11450094; doi:10.1038/s41420-024-02152-7)

Figure 2C

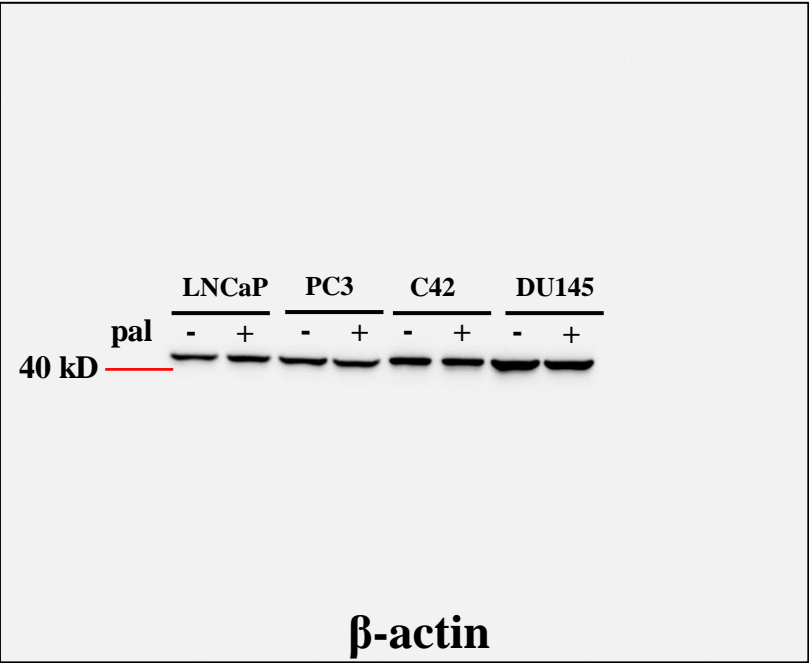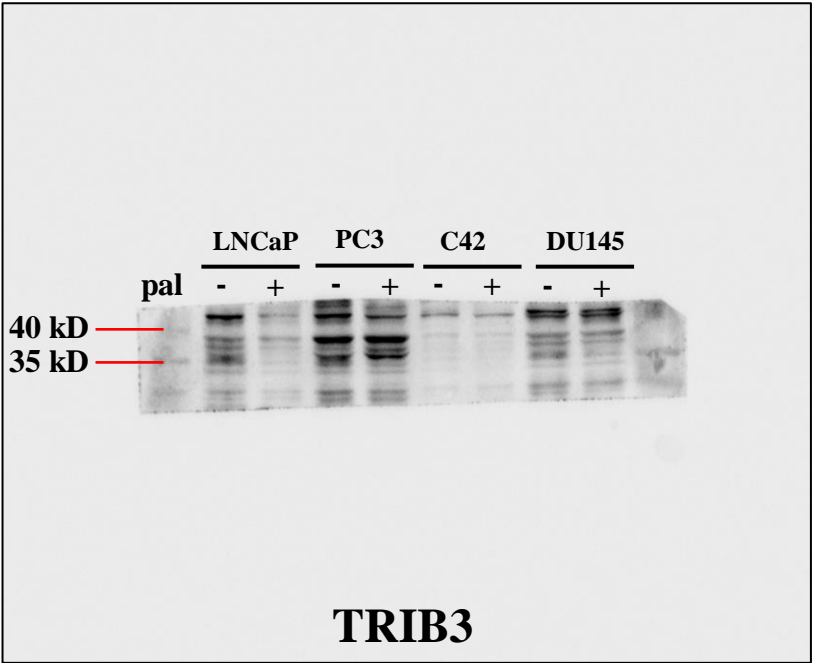

Figure 5F

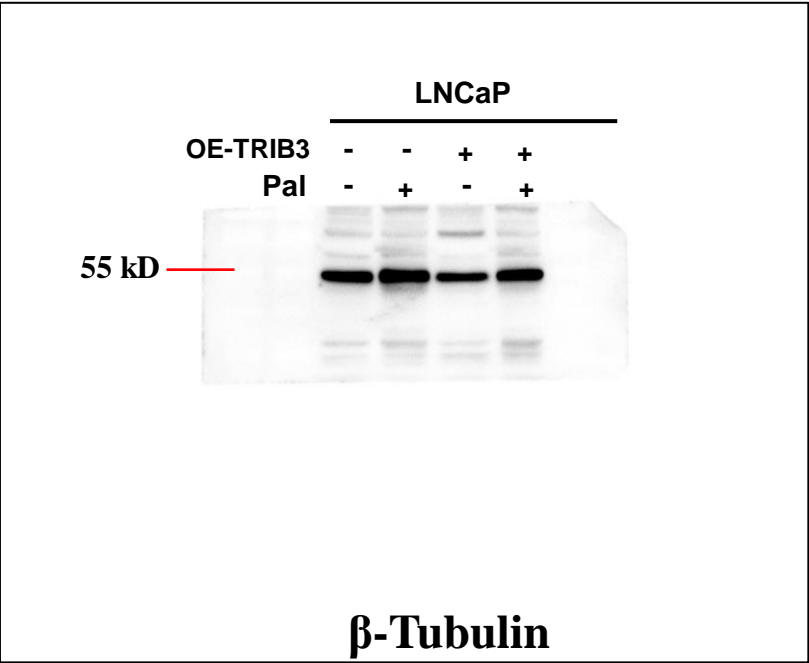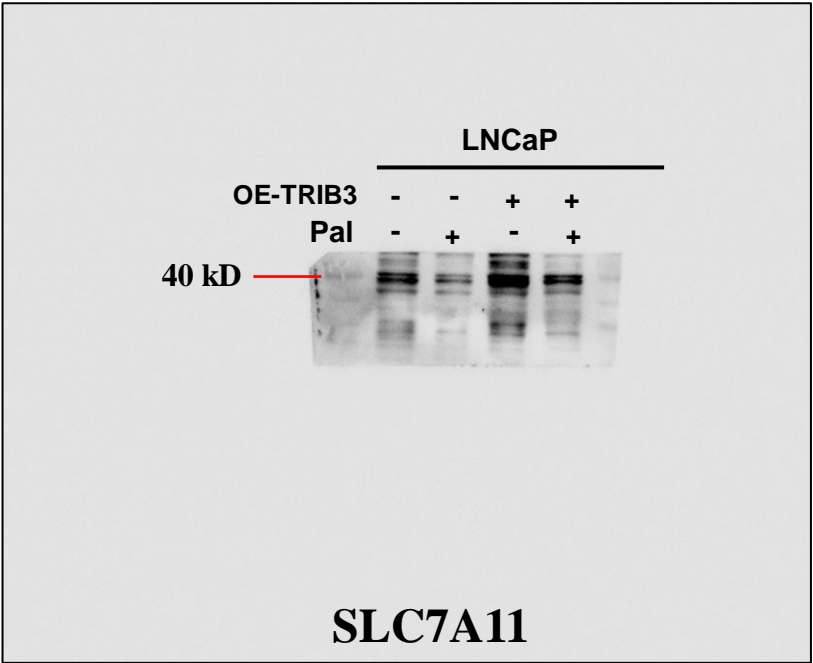

Figure 6A

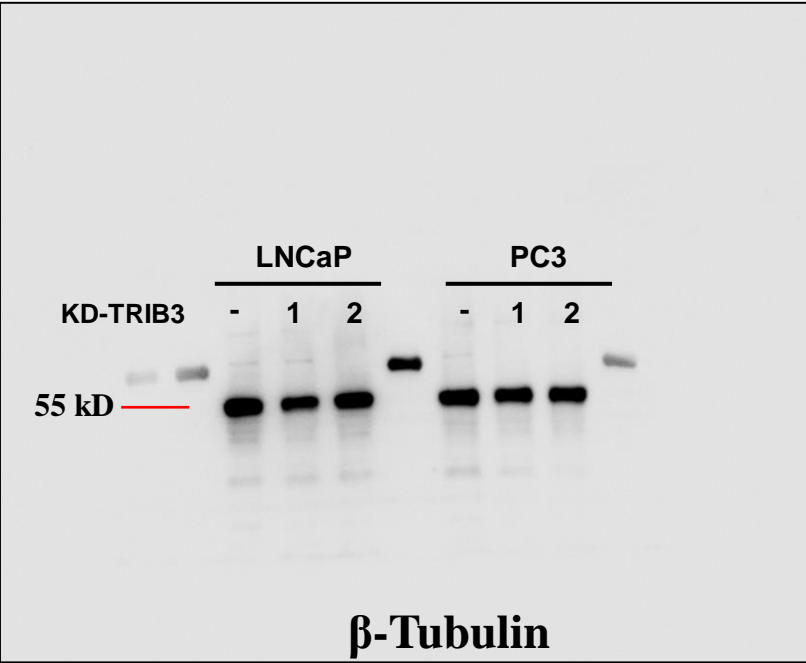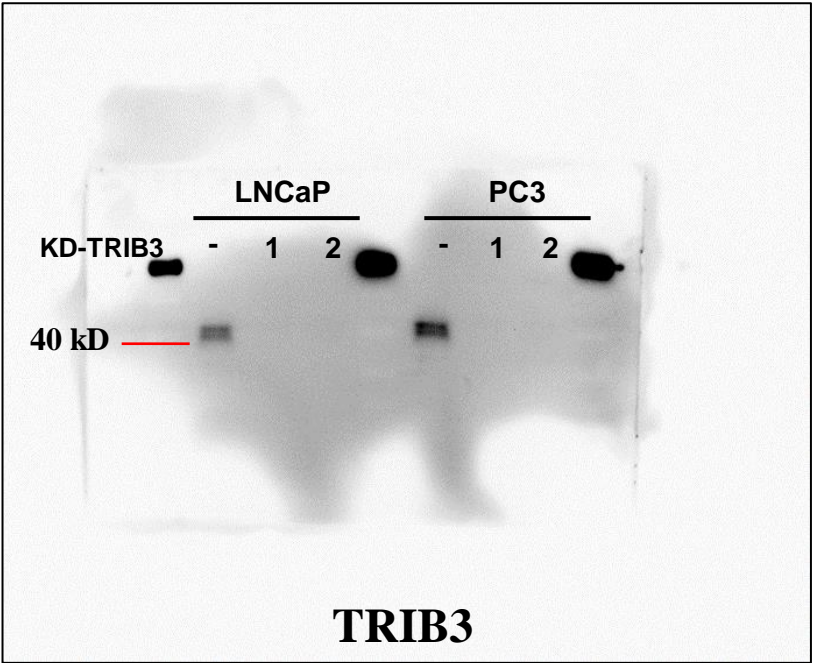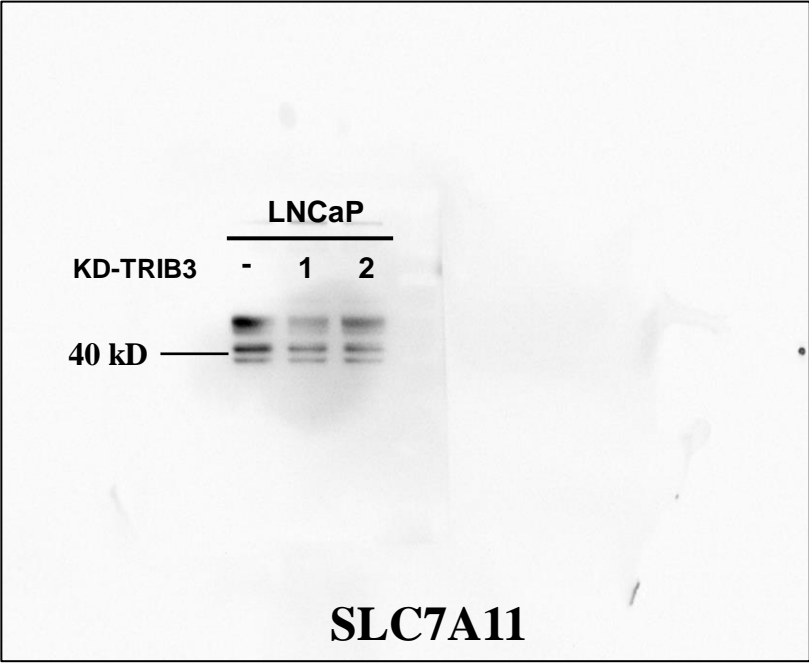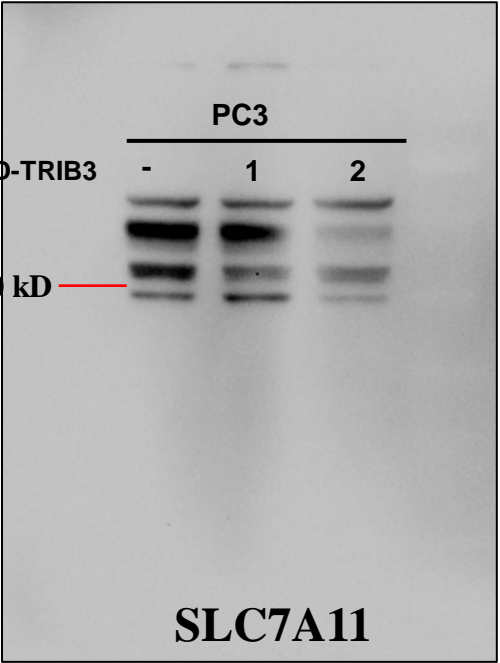

Figure 6A

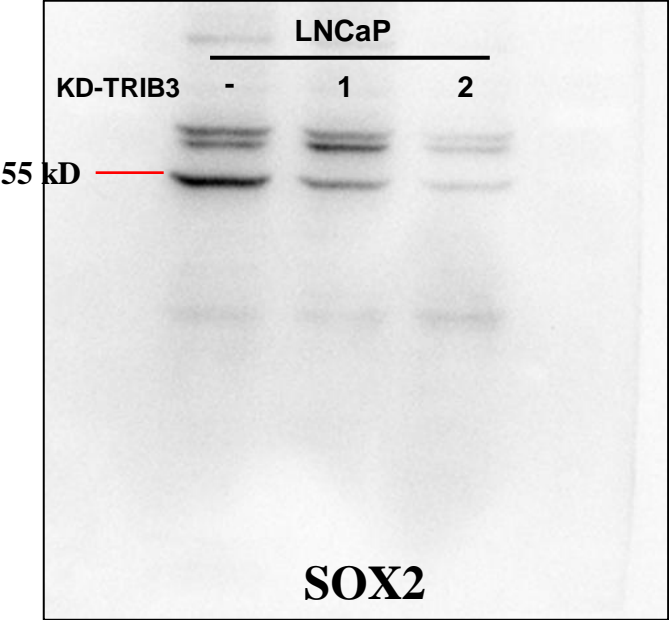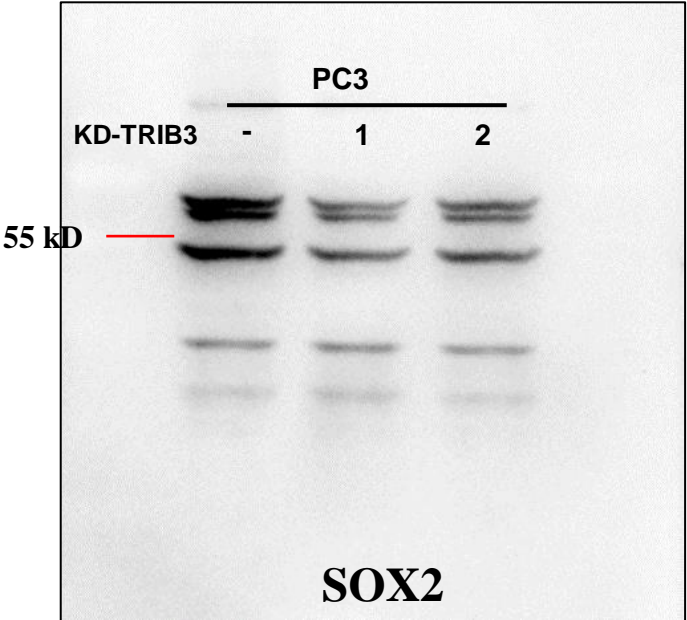

Figure 6B

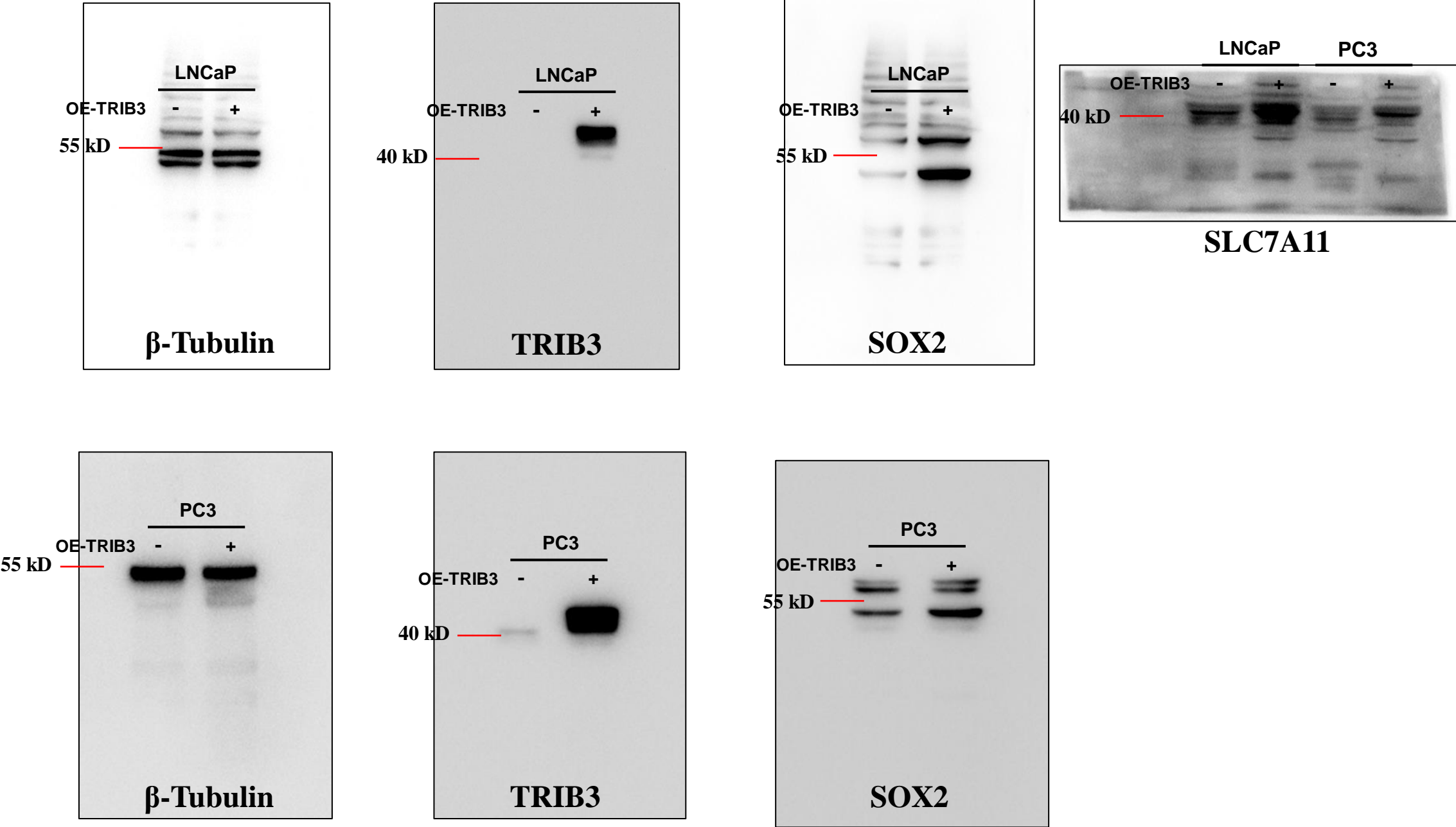

Figure 6G

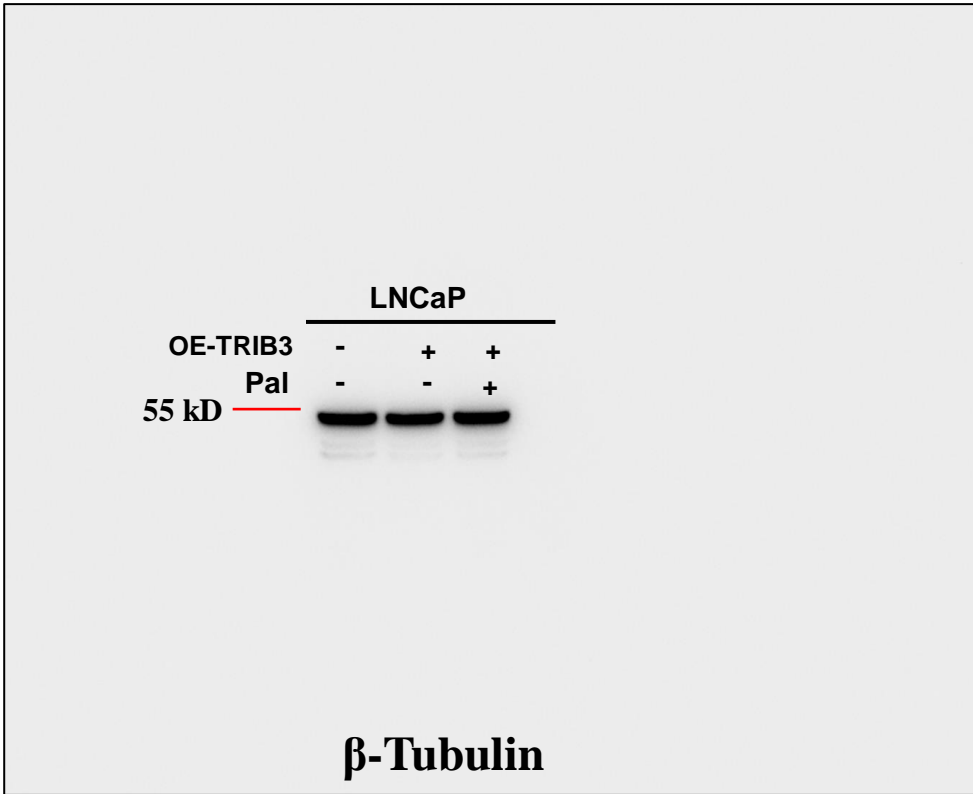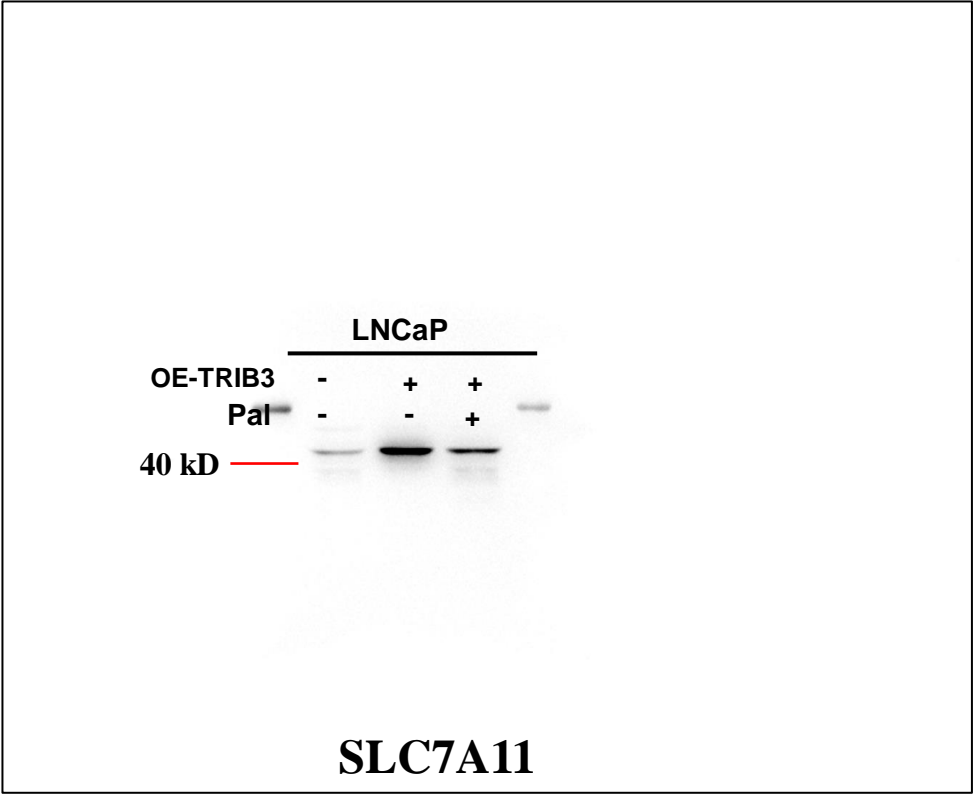

Supplement: Supplementary file 3 — Original Data File [file 41420_2024_2152_MOESM3_ESM.pdf]
